# An Analysis of Canadian Psychiatric Mental Health Nursing through the Junctures of History, Gender, Nursing Education, and Quality of Work Life in Ontario, Manitoba, Alberta, and Saskatchewan

**DOI:** 10.1155/2013/184024

**Published:** 2013-04-28

**Authors:** Mary Smith, Nazilla Khanlou

**Affiliations:** ^1^Queen's University, Kingston, ON, Canada K7L 3N6; ^2^School of Nursing, Faculty of Health, York University, 4700 Keele Street, Toronto, ON, Canada M3J 1P3

## Abstract

A society that values mental health and helps people live enjoyable and meaningful lives is a clear aspiration echoed throughout our Canadian health care system. The Mental Health Commission of Canada has put forth a framework for a mental health strategy with goals that reflect the virtue of optimal mental health for all Canadians (Mental Health Commission Canada, 2009). Canadian nurses, the largest group of health care workers, have a vital role in achieving these goals. In Canada, two-thirds of those who experience mental health problems do not receive mental health services (Statistics Canada, 2003). Through a gendered, critical, and sociological perspective the goal of this paper is to further understand how the past has shaped the present state of psychiatric mental health nursing (PMHN). This integrative literature review offers a depiction of Canadian PMHN in light of the intersections of history, gender, education, and quality of nursing work life. Fourteen articles were selected, which provide a partial reflection of contemporary Canadian PMHN. Findings include the association between gender and professional status, inconsistencies in psychiatric nursing education, and the limitations for Canadian nurse practitioners to advance the role of the psychiatric mental health nurse practitioner.

## 1. Introduction

The account of the way Canadian psychiatric mental health nursing (PMHN) has emerged into its current state can provide an insightful perspective that fosters a better understanding of its present challenges and opportunities. This review paper examines the development of Canadian PMHN in Ontario and the western provinces since the beginning of the 20th century. Canada consists of 3 territories and 10 provinces. With a population of approximately 34,880,500, Canada had the highest growth rate of the G8 countries in the 2011/2012 period [1].

In Canada, registered nurses (RNs), registered psychiatric nurses (RPNs), licensed practical nurses (LPNs), and nurse practitioners are employed in the area of psychiatric or mental health care. RPNs are a regulated separate profession in the provinces of Manitoba, Saskatchewan, Alberta, and British Columbia (For the purposes of this paper RPN refers to registered psychiatric nurses that are only legislated in the four western provinces. In Ontario, registered practical nurses, also abbreviated to RPN, provide care in all settings and are not solely specific to or trained in the area of psychiatric mental health. When RPN is used in this paper, the reference is to registered psychiatric nurses.)

Statistics on the various types of nursing professions employed in psychiatric or mental health care can reflect pertinent conditions in relation to the Canadian mental health system. For instance, in 2010, 5.1% of all RNs in Canada were working in the mental health care sector, a slight decrease from 2007 where 5.2 were employed in this same area [2]. In Ontario alone, between 1993 and 2003, there has been a 29.4% drop in the number of nurses working in the psychiatric sector [3]. The number of RPNs increased by 3.8% since 2007 but the RPN workforce remained at 1.5%. The proportion of full time RPNs in psychiatric nursing decreased from 67.8% in 2007 to 62.2% in 2011. The RPN profession also has the highest percentage of males, 22%, of all the Canadian nursing professions [2]. According to the Canadian Nurses Association, nurse practitioners (NPs) account for 0.9% of the entire number of RNs [4]. Of the 2,486 NPs employed in Canada, 1,482 or 60% work in Ontario. The percentage of NPs working in psychiatric mental health in 2010 was 1.4% [4].

The statistics concerning the employment of nurses in the mental health care sector are meaningful when considered in light of the statistics concerning how many people in Canada suffer from mental health problems. The Canadian Community Health Survey by Statistics Canada was completed in 2003 and is scheduled again in 2013. This will enable further comparisons of how Canada fairs in terms of prevalence of mental health disorders and the mental health services available [5]. In 2003, it revealed that two-thirds of those who experience mental health problems do not receive mental health services [6]. Furthermore, persons living with mental illness may have difficulty competing for limited health care due to high rates of poverty and disability [7]. In 2012, the Institute for Clinical Evaluative Sciences and Public Health Ontario released the report “*Opening eyes, opening minds: the Ontario burden of mental illness and addictions report”* [8]. This report measures the extent of mental illnesses in Ontario in comparison with the prevalence of other medical conditions. Addictions and mental illnesses represent an enlarging burden. The need for improved accessibility of mental health services is emphasized [8]. The size of the PMHN workforce and the accessibility of services may impact mental health care for Canadians.

Historically, the deinstitutionalization trend that began in the 1960s resulted in many persons with mental illness being released into the community where a serious lack of mental health services prevailed [9]. This led to a significant portion of individuals with mental illness to become imprisoned in jails or detention centers with limited access to mental health care. Additionally, a revolving door phenomenon occurred where previously institutionalized individuals were readmitted into acute psychiatric care settings [9].

To further understand the Canadian context for PMHN, it is useful to be familiar with developments concerning mental health care in Canada. There are recent major initiatives underway in Canada with regards to mental health reform. In 2005, a national review of the Canadian mental health system took place as outlined in the Kirby report [10]. In 2006, the Standing Senate Committee on Social Affairs, Science and Technology completed the final report entitled “*Out of the shadows at last—transforming mental health, mental illness and addiction services in Canada” *[11]. As the mental health care system was considered to be fragmented, the final report made recommendations for the reformation of mental health care. The assembly of a Mental Health Commission to enable a national strategy for mental health care was emphasized by Michael Kirby, the chairman of the Standing Senate Committee on Social Affairs. Funding for the commission came in 2007, and in 2009, the Mental Health Commission of Canada released the framework “*Towards recovery and well-being: a framework for a mental health strategy for Canada” *[12]. Seven goals are set in this framework that represent how a transformed system will appear. The goals depict that everyone should have the opportunity for optimum mental health and well-being [12]. The Mental Health Commission has recognized that there is a lack of opportunities for Canadians with mental health disorders to achieve optimum mental health. This calls for key stakeholders, that is, groups interested in responding to people with mental health concerns, to address ways to further the provision of mental health care. Nurses are the largest group of healthcare providers, and nurses who provide mental health care are important stakeholders in meeting the mental health care needs of Canadians.

In 2012, the Mental Health Commission of Canada released the blueprint “*Changing directions, changing lives: the mental health strategy for Canada”* [13]. Six strategic directions are given in this report including the provision of mental health services and treatments accessible to the people who need them. Reference to the vital role nurses have in conjunction with the team of health care providers to fully achieve the strategic directions is not explicit in the 2012 report. Community health nurses are instrumental in providing mental health care in the community. In addition, NPs are becoming increasingly more common in providing primary health care within Canada. Their role in the assessment, diagnosis, and treatment of mental health disorders may be extremely useful to further the strategic direction concerning enhanced accessibility to treatment. Collectively, nurses and nurse practitioners have a critical role in the strategic direction as identified by the Mental Health Commission of Canada. A national strategy is needed, and mental health care providers will need to be included in this strategy according to the Kirby report [10]. Nurses who form the largest group of health care workers in Canada may need to be strong stakeholders in the strategic direction for mental health care in Canada. The commission has yet to clearly voice how health care and nurses will be reorganized to support accessible services and treatments.

Kirby [14] indicates the pivotal position that mental health care providers have and also addresses concerns regarding the stress levels and mental health of health care professionals themselves. Indeed this leads to concerns regarding the quality of nursing work life for PMHN and how this specialty is responding to the demands of the mental health care system. The analysis of the history of Canadian PMHN can enable a better understanding of why PMHN is the way it is today. With this understanding, opportunities for further PMHN development, education, and future research may surface.

Given the need for increased accessibility to mental health care and treatment, it is helpful to further explore how nursing will move to foster improved mental health care. At this time, there is a shortage of research studies that look at how the current nursing workforce specifically addresses improved accessibility to mental health care. Career pathways to become NPs that specifically work in mental health are lacking, where NPs may diagnosis and treat mental health conditions. As an NP working in mental health care, the options for pursing advanced mental health nursing education in Canada are limited. PMHN education varies across Canada and unlike the United States there is no legislated psychiatric mental health nurse practitioner (PMHNP) role. Also RPNs may wish to become advanced practice nurses or PMHNPs that are able to diagnose or prescribe psychotropic medications. A regulated PMHMP's role may further the accessibility of mental health care in Canada. There are opportunities for nurses to achieve a master's degree in mental health in Manitoba; however, there are no programs in Canada that specifically yield PMHNPs that may diagnose or prescribe medications particular to mental health care. The educational programs for nurses also vary in each province in terms of content and the prerequisites. With the variety of different educational approaches to PMHN in Canada, the career path to advance nursing practice in PMHN is complicated. An RPN in the western provinces has had different preparation than an RN working in mental health care in Ontario. How these diverse PMHN educational approaches have come to be and their implications for Canadian PMHN can provide useful insights to inform future directions to PMHN in Canada.

## 2. Materials and Methods

### 2.1. Review Aims and Research Question

The purpose of this inquiry is to understand the current state of mental health nursing in Canada through the intersections of history, gender, nursing education, and quality of work life as evidenced by the existing research and literature. The research question that guided it was as follows: what does the research reveal about contemporary Canadian PMHN in terms of the junctures of history, gender, education, and quality of PMHN work life? 

An integrative methodology was determined to be most suitable for this review due to the scarcity of data on the organization and efficacy of Canadian PMHN. Rigor was maintained through evaluating primary sources for methodological quality. The integrative review includes the stages of problem identification, literature search, data evaluation, data analysis, and presentation.

### 2.2. Integrative Review

Whittemore and Knafl [15] explain that the integrative review is particularly suited to inquiries with limited existing empirical research. Historical events in PMHN are relevant to how this field has come to be. Yet few research studies exist on Canadian PMHN, and the studies are in the form of social historical analyses that reflect on the events of the past. Moreover, there is a paucity of data pertaining to Canadian NPs specific to mental health care and NP mental health education. Sociological, critical, and social historical analyses, reports, and surveys on Canadian nursing and Canadian mental health care offered insight into the field of Canadian PMHN that furthered this inquiry (see Tables 1, 2, and 3). In the integrative review, according to Whittemore and Knafl [15], diverse methodologies may be incorporated to further a comprehensive grasp of issues suited to the complexities of health care. Both quantitative and qualitative research may contribute to the perspective concerning a phenomenon. The culmination, analysis, and evaluation of diverse data and research regarding PMHN enabled a perspective that may begin to further understanding mental health care in Canada in light of the lacking research solely pertaining to Canadian PMHN or PMHNPs.

### 2.3. Theoretical Perspective

A sociological perspective allows for a critical assessment of common assumptions and fosters recognition of opportunities and constraints that shape our circumstances, the interplay between societal forces and personal lives, and of human diversity [16]. In addition, the concept of gender stratification is comprehensively analyzed within sociology and is applicable to the topic of this paper. Gender stratification concerns the imbalanced division of privilege and power between females and males [17]. A sociological perspective is appropriate for this inquiry as nurses are employed within gendered hierarchal structures that interact and are influenced by the larger political context.

Feminist theories, emerging from a sociological perspective, address patriarchy, power structures, and gender inequality. They are concerned with the societal organization and interactions that maintain male authority and female subordination [18]. A feminist perspective enables the depiction of the social structures leading to the devaluation of women. Social order then becomes the problem rather than women themselves [18]. The intent in this work is not to devalue women or nurses but, rather, to illuminate the social structures that have persevered throughout history and have influenced PMHN education and quality of work life. The sociological perspective applied here encompasses feminist concepts.

A sociological perspective also bridges history and how things are today. Wright [19] calls this way of thinking the sociological imagination that grasps the connection between history and the way we are and the way we are becoming. The history of PMHN seen through a sociological perspective can promote an understanding of its current state.

### 2.4. Search Strategies

The primary literature search involved databases, the internet, and published online journals. The databases included CINAHL, PsycINFO, Evidence-Based Mental Health, Cochrane, PubMed, and ProQuest. Published online journals were accessed through Blackwell Synergy and Sage. Primary search results were then analyzed to search their references for secondary sources of interest. The databases were accessed through the York University Library, Toronto, Ontario and Queens University Library, Kingston, Ontario. Texts were borrowed through the assistance of the librarian at Waypoint Centre for Mental Health Care, Penetanguishene, Ontario.

Through combinations of keywords, topics searched for included psychiatric nurses in Canada, Canadian psychiatric nursing history, Canadian psychiatric nursing education, Canadian mental health nursing, Canadian psychiatric mental health nursing, Canadian nurses and gender, quality of life for Canadian psychiatric mental health nurses, stress experienced by psychiatric nurses in Canada, registered psychiatric nurses in Canada, work stress of nurses working in mental health, literature review on psychiatric mental health nursing in Canada, sociological perspective of psychiatric nursing, psychiatric mental health nurse practitioner, mental health nurse practitioner education, and sociology of nursing stress.

The searches included English language reports, studies, reviews, editorials, and narratives and was limited to Canadian sources and Canadian PMHN. The intent was to better understand the Canadian context of PMHN. International comparisons were beyond the scope of this undertaking. With regards to the unique Canadian historical influences shaping PMHN, 14 studies were selected as relevant. There was a lack of studies that focused solely on the Canadian PMHN experience as many reports included RPNs and RNs. RNs in the provinces east of Manitoba do not have a separate psychiatric regulation, as do the western provinces. No studies were found on the education in Canada for NPs in mental health. As a result data often combines all nurses. This made it difficult to discern the unique state of Canadian PMHN from that of all Canadian nurses.

## 3. Results

The results from the literature review are divided into three sections. The first section reviews the literature from 2 sources that include a critical sociological perspective. The next section considers the interrelation of history, gender, and the development of education for Canadian PMHN through the review of 6 social, historical, and analytical studies. The final section focuses on the quality of Canadian mental health psychiatric nurses work life from 6 survey reports.

### 3.1. Studies with a Sociological Perspective Relevant to PMHN

Wall [20] utilized a sociological outlook, as described in Section 2.3, to critique nursing practice settings and nursing research. The experiences of nurses can be further understood through a lens that encompasses professionalization and organizational influences. In addition, gender is ingrained in all of nursing where a patriarchal culture manifests. According to Wall [20], the control of medicine over nursing is evident in nursing's utilization of quantitative approaches in research that is consistent with the history of medicine. Health care, being a largely institutionalized entity, has its own way of socializing nursing and continues to place medicine at the apex of health care despite health care reformation. Conversely, Wall [20] does not comment on the socialization or culture of medicine with regards to gender, as medicine being once an overwhelmingly male profession has increased the number of female practitioners. Furthermore, the discussion by Wall [20] discusses nursing in general and does not specify particular sectors of nursing such as NPs. Nevertheless, a critical sociological paradigm may help to further understand issues in nursing and PMHN related to knowledge, gender, professionalization, and organizations [20].

McGibbon et al. [21] utilized Smith's [22] sociological frame of institutional ethnography to reconsider the stress in nursing through interviews, focus groups, and observation of pediatric intensive care nurses [21, 22]. Results suggest that stress is framed within the social structure of organizations that may have hierarchical and power-based relations. Articulating patient matters may be challenging in organizations with hierarchical systems. This may contribute to the level of stress nurses experience. Studies concerning nursing stress or vicarious trauma often neglect how gender influences the life of nurses. Theories that are utilized in nursing research may avoid gender analysis. Without a gendered analysis, occupational stress may not be illuminated or solutions may not be identified. Nursing may further benefit by utilizing a gender perspective in research to potentially unveil social patterns that may influence teamwork and collaboration. Gender, race, and class are aspects of nurses' identities but may not be within the range of the theory utilized for the research. This may lead to research that may neglect identification of concerns for nurses [21]. The critical gendered and sociological perspectives as depicted by Wall [20] and McGibbon et al. [21] provided the lens in how PMHN is perceived in relation to the following results.

### 3.2. Intersections of History, Gender, and Education for Canadian PMHN

Tipliski [23] contributes to the understanding of Canadian PMHN history in the study entitled “*Parting at the crossroads: the emergence of education for psychiatric nursing in three Canadian provinces,” 1909–1955. *Gendered roles may have permitted psychiatry to maintain control of psychiatric nursing education in the western provinces. Unlike Ontario, where nurses were able to assume control over PMHN, thereby allowing PMHN incorporation into general nursing education, the provinces of Manitoba and Saskatchewan permitted psychiatry to prevent the merging of PMHN with general nursing. From here, as Tipliski explains, two different models for Canadian PMHN education developed leading to the class of the RPN that only exists in the western provinces [23]. This may have fostered a partition in the PMHN nursing force in Canada, as the RPN designation presents a separate regulation of nurses that is nonexistent east of Manitoba.

Tipliski [23] describes how the nursing leaders of Saskatchewan and Manitoba failed to assert control over nursing education. In 1955, the Canadian Nurses Association (CNA) recognized how the separate training that was occurring in the western provinces was not conducive to the efforts to professionalized nursing for all of Canada. Unfortunately, a progressive movement by the nursing leaders of that time to bring psychiatric nursing under the umbrella of general nursing in the 1950s dissolved. This left the western provinces with a split nursing educational approach where psychiatric nurse training remained separate from general nursing education and in the hands of psychiatrists [23]. Although psychiatric nursing is no longer controlled by the psychiatrists in the western provinces, separate training for RPNs remains. From this perspective, it can be appreciated how female nursing leaders in Ontario influenced historical developments to integrate psychiatric nursing education, which was consistent with earlier efforts made by CNA to converge the psychiatric nursing education with general nursing in the western provinces. Ontario nurse leaders were able to gain control over their own nursing education and practice and were aided by the government. Tipliski [23] explains how the Ontario male medical superintendents attempted to keep mental nursing separate from general nursing thus hindering the professionalization of nursing. The contributions from Nettie Fidler in 1933, a nurse graduate from the Toronto General Hospital, together with a report by Professor George Weir that recommended the merging of general with mental nursing, stimulated the progression by the Registered Nurses Association of Ontario (RNAO) to advocate for closure of the separate schools providing only mental health training [23]. It is possible that this account may overlook some of the other possibilities accounting for how PMHN developed in diverse ways, yet Tipliski [23] provides a description of the history of PMHN that may help to explain how gender dynamics influenced the development of PMHN in Canada. Despite the variation between educational approaches that resulted in the RPN designation for the provinces east of Ontario, there is a lack of evidence revealing how PMHN care varies given the differing education preparations.

Of interest is Tipliski's [23] reference to how gender may have been a factor in the development of the RPN designation of the western provinces. This relates to the feminist concepts of patriarchy. Brown [18] defines patriarchy as a social system that holds several assumptions. One assumption portrays women as being assigned a social function. In nursing, as Tipliski [23] depicts nurses were assumed to be fitting to provide care due to their female gender with their inherent ability to nurture. The view of female nurses as nurturers may have been a patriarchal belief held by the male medical superintendents. Another patriarchal assumption is that women are thought of as weaker and less strong. Tipliski [23] also considers the patriarchal context of PMHN, where the nurse leaders of the western provinces may have struggled against the authority of the medical profession. On the other hand, Tipliski [23] does not speak of the power issues between general nursing and medicine or the professionalization the regulated psychiatric nurses of the western provinces experienced.

Dooley [24] argues that the separate class of psychiatric nurses in Manitoba developed their own unique craft that is specialized for the mentally ill population. In this study, the account from Manitoba mental health nurses of the 1930s supports the view that the development of their nursing profession was the outcome of their cooperation with physicians. Female mental health nurses in Manitoba considered themselves skilled and at a higher level than the male attendants. The female nurses were often in supervisory positions directing the personal care given by the male attendants. This contrasts with Tipliski's [23] view on the separate division of psychiatric nursing being related to paternalistic structures and in which female mental nurses could not overcome male physicians and psychiatrist's domination that sought control and power. Yet the Manitoba mental health nurses that would become RPNs asserted their distinct class, and this has enabled the continuation of the separate training for mental health nursing that continues to manifest inside the western provinces. Evidence to suggest that the separate divisions of psychiatric nursing education in Canada that have any effect on the quality of mental health care has not been substantiated in research.

One must also acknowledge the circumstances that influenced Canadian women as nursing leaders in the past. For instance, Dooley [24] describes the social context of the interwar years where women were looking for ways to support themselves, and mental health nursing provided a way to live that would provide regular meals and housing for mental health nurses. From this description, it is possible that PMHN also developed in diverse ways in Canada as a result of social and economic circumstances in addition to gender influences, that have yet to be fully explored.

Hicks [25] provides further details concerning Manitoba's adoption of the RPN model. Through a genealogical analysis, the study considers historical circumstances that led to the RPN model. Gender stands out as a main influence in this movement where the male leaders of the psychiatric nursing associations of the adjacent western provinces were influential in drawing Manitoba to call for the distinct nursing class. The male nurses of the RPN psychiatric nursing associations in the western provinces developed a collegial relationship with the male attendants of Manitoba who sought RPN status. This presents an interesting insight with regards to gender, in that the men were able to increase their strength and power through the joining with the male psychiatric nurses of Manitoba. This also diverges form Tipliski's view where the psychiatric nurses were submissive to medical authority in relation to the female gender. In addition, the separate psychiatric RPN distinction was favored by the male medical superintendents as there was a lack of interest by the general nurses to work in psychiatry [25], which may have also furthered the movement towards the RPN class. Hicks [25] considers the large number of male attendants and the significance of gender in the creation of the RPN designation. Male attendants provided custodial care and sought to provide nursing care that would elevate the status of the male attendants. Unlike Dooley [24] and Tipliski [23] who focus on the female gender of nursing, Hicks [25] depicts the collegiality and support of male RPN leaders of the western provinces. The Canadian Council of Psychiatric Nursing provided support to the Manitoba attendants who sought RPN status [25]. The RPN emergence may be seen as a way for the male gender to enter into nursing in a time where nursing was culturally enshrined as a female role and the attendants of psychiatric institution sought status and class through the RPN profession. In this way, Hick's study demonstrates how male gender has influenced the history of Canadian PMHN [25].

Boschma et al. [9] examined nurses' stories that further the understanding of the development of PMHN in Alberta. RN status was recognized as being desirable for PMHN and could be achieved by mental health nurses by taking an extra 18 months of training in a general hospital after completing 2 years in a psychiatric hospital. Women were sought as nurses by the governing psychiatrists for their caring and compassionate nature. The male attendants were excluded from nursing because of their gender and became increasingly resentful. The male attendants sought recognition and professional status. Like Saskatchewan, the separate status from RNs was lobbied for by the male attendants and in 1963 registration was given to psychiatric nurses [9]. In this sense, the development of the separate RPN status stems from the gender division that favoured RN status to females leaving male mental health attendants to seek professional status by becoming RPNs. Similar to Hicks [25], Boschma et al. [9] also indicate the influence of male attendants. This differs from the view held by Tipliski [23] that mainly denotes the oppression of largely female nurses by the medical superintendents. Likewise, the account by Hicks [25] and Boschma et al. [9] also varies from the perspective by Dooley [24]. Dooley [24] explains that the female psychiatric nurses saw the RPN category as separate and offering more to the mental health care of patients than the general nurse education could offer. Despite the development of the RPN class, mental health care in Alberta continued to suffer and the need for further education continued pressure to achieve RN status [9].

Professionalization through the regulation of RPNs is described further by the interweaving of data from Boschma's case study of community mental health in Alberta [26] and Hick's historical review of psychiatric nursing in Manitoba [27]. Boschma [26] explains that Alberta graduates from the mental hospitals that started in the 1930s met with resistance from the general nursing organizations leading to the formation of the separate professional organization for RPNs. Hicks [27], consistent with Tipliski [23], indicates that the Alberta Association of Registered Nurses (AARN) had opposed the plan by superintendent Charles Baragar to establish a program to train psychiatric nurses. The AARN recommended that general nurses may complete a postgraduate psychiatric course or combine with general nursing students during a third year of training. They also suggested the hiring of general nurses who were unemployed. Baragar, however, was able to succeed in his intentions through his appeal to the minister of health, and the program was implemented. This program included a total of 4 years of instruction at the mental hospital with 2 of these years occurring at the general hospital. Male attendants were restricted from attending and had to complete a three-year psychiatric program. The male attendants went on to successfully lobby for the Alberta psychiatric professional nursing association [27]. Hicks [27] explains that the association's activities during that period may have been more union type activities rather than professional. In the 1950s, the movement to form the psychiatric nursing association was developing in Manitoba. The Canadian Nurses Association recommended combining the RN and psychiatric training programs in disapproval of the two models emerging for psychiatric nursing in Canada. Despite efforts to organize this endeavour, Canadian Nurses Association's (CNA) plans were never realized [27]. Boschma [26] and Hicks [27] further explicate that beyond the development of the RPN profession to meet the poor staffing of the mental health facilities, the profession of psychiatric nursing in the western provinces grew to deliver a much needed service that provides specialized mental health nursing differing from what general nursing could supply. The RPN profession may advance mental health nursing knowledge and care through the experiences gained from this profession's unique historical development.

### 3.3. Quality of Canadian Mental Health Psychiatric Nurses Work Life

In 2001, the Canadian Nursing Advisory Committee (CNAC) formed as a result of the recommendations by the Advisory Committee on Health Human Resources (ACHHR). The ACHHR's first recommendation of the *Nursing Strategy for Canada* [28] was to create the CNAC. The CNAC included nursing representatives from the three nursing professions of RNs, RPNs, and LPNs. The CNAC's main goal concerned the quality of nursing work life and the identification of provincial and territorial strategies to enhance nursing work life. With the shrinking workforce this was deemed a high priority. The CNAC's recommendations concern all nursing workplaces including settings that provide PMHN.

The CNAC commissioned 6 projects which looked at strategies to improve workplaces, costs related to absenteeism and overtime, workload issues, satisfaction of nurses in the workplace, workplace respect, and autonomy and health care organizational structures [29]. As a result, 51 recommendations were made to the ACHHR. The recommendations can be summarized into three categories. The first category concerns management issues and resources. There is a need to reduce nursing's pace and intensity, increase full time work, decrease sick time and overtime, and enable full scope of practice. The second recommendation speaks to professional work settings that foster a thriving and dedicated workforce. Respect for nurses is key to this recommendation, as well as education at the master and doctoral levels. Education for nurses should be accessible in remote and rural areas. Violence and abuse in the workplace must be addressed. In the third recommendation, monitoring of the health of nurses and workplaces and disseminating information to keep nurse abreast of initiatives and education are considered. Accreditation, awards to promote quality of nursing work life, continued research on the nursing workforce, implementation of regional nursing committee recommendations, national nursing retention, and recruitment campaigns with a heightened emphasis for diverse and aboriginal groups are a few activities mentioned within this recommendation [29].

The recommendations strive to rectify the main issues that concern the shortage of nurses, the lack of educational opportunities, the limited scope of nursing practice, and the unfavourable working conditions, which are also applicable to the RPNs and RNs who provide PMHN. However, the CNAC recommendations lack consideration of gender and its intersection with professionalization and workplace culture that culminate in the core of issues, including patriarchal culture- and power-based hierarchical organizations that impact nursing work lives [20, 21]. Lacking also from the CNAC recommendations is reference to PMHN being a stakeholder in mental health. As a stakeholder, PMHN needs to organize as a united front, so that its voice is heard. This may be challenging as more powerful stakeholders, like medicine, have traditionally dominated health care [20, 21].

Maslove and Fooks [30] conducted a study to determine the degree of implementation of the 51 CNAC recommendations made in 2002 as requested by the Office of Nursing Policy at Health Canada. Policies of the stakeholder organizations were assessed to determine their impact on facilitating the implementation of the recommendations. Their methodology included scanning of websites, sending letters to 94 stakeholders to determine their progress, and interviewing 14 informants to identify barriers and supports. The 94 stakeholders included employer organizations, the federal government, provincial/territorial governments, unions, professional associations/regulators, educators, research community, and national organizations. There was a 50% response rate from stakeholders. Findings were then shared with nursing stakeholders at a roundtable to enable feedback [30].

The recommendation to increase the number of education seats occurred in a uniform manner. However, other recommendations occurred in some areas but not all, and there was difficulty in determining what had occurred nationwide. Implementation of the recommendations including workload measurement systems, increased full time positions, analyzing sick time, increasing nurse mentors, and flexible scheduling were not consistently taking place throughout Canada. Key informants favoured regulation at the provincial versus the national level. The lack of stable funding was depicted as a barrier to implementation of the recommendation to develop secure jobs. The varying professional associations and regulatory colleges that exist in each province may complicate assigning recommendations [30]. Respondents addressed lack of interest from government regarding nursing issues. Accountability is seen as critical to implementation of the recommendations made by the CNAC. Determining what organizations should be responsible for is a priority concern, and employers need support to enable improvements that will impact nursing quality of work life [30]. Organizations may require government funding in order to implement the recommendations that will enhance nursing work environments.

Together, the CNAC's recommendations [29] and the study by Maslove and Fooks [30] include data from RPNs and general nurses who also provide psychiatric care in the provinces east of Manitoba. Issues pertaining to violence and abuse were seen as priority issues [30]. Poor working conditions may negatively impact quality care, and the lack of action and accountability by organizational and provincial territorial leaders and their support to employers [30] may adversely impact the mental health of both nurses and the Canadian population, which they serve.

*Findings from the 2005 National Survey of the Work and Health of Nurses* (NSWHN) [31] examined the health of Canadian regulated nurses as related to their work environment. The data as presented here was collected between 2005 and 2006. Nineteen thousand nurses inclusive of RNs, RPNs, and LPNs were surveyed with a response rate of 80%. More than one-quarter reported being physically assaulted. Of note is that 44% of males reported assault compared to 28% of female nurses. The reasons for this finding are not elaborated upon in the 2005 NSWHN. It is not known if this points to male nurses being more likely to be assaulted or more likely to report assault. Forty-four percent of nurses reported emotional abuse. High physical demands were reported by 75% of LPNs, 60% of RNs, and 45% of RPNs. Gender difference was not specified for physical demands, that is, if male or female nurses reported higher physical demands. The mental health of nurses was adversely associated with evening shifts and employment in long-term care facilities. Lack of respect and low support from coworkers and superiors were linked with poorer mental health. In addition, the mental health of nurses was also affected by elevated job strain, low autonomy and control, and poor physician relations. One in ten nurses reported having depression and needing to take time off in relation to their mental health. The finding of depression in nurses is compared with the overall employed population that utilized data from the Workplace and Employee Survey, Labour Force Survey, and the Community Health Survey [31]; however, comparisons with specific groups or professions are not explicit. Of all nurses, including both male and female, 9% experienced depression. The experience of depression by sex was not included for nurses but for all; employed the 4% of men and 7% of women experienced depression. Also at the time of this study in 2005, one-fifth reported their mental health difficulties interfering with their jobs. Quality of care was negatively affected by inadequate staffing. Thirty-eight percent of nurses felt that staffing was inadequate. Improvements in quality care were related to improved management and more staffing [31]. The findings of this survey may further illuminate the working conditions for nurses and the implications for their mental and physical health.

The Health Policy Research Bulletin (HPRB) is published usually twice yearly by Health Canada with the purpose of reinforcing the evidence that supports decision making in health policy. In 2007, the HPRB's issue, titled “*The working conditions of nurses: confronting the challenge” *[32], focused on the Canadian nurses' working conditions and implications for the country's health care system. Between 1997 and 2005 overtime by RPNs, LPNs, and RNS increased in all areas where nurses work by 58%. In light of the high overtime rates, it is questionable if the current level of full-time nursing positions in mental health care in Canada is sufficient to adequately care for those with mental illness. All areas in nursing face similar pressures concerning increased overtime and a need for more full time work; however, mental health settings entail frequent interactions with challenging and difficult behaviours. This may intensify the stress on nurses working in mental health care where there is shortage of full-time nurses.

Robinson et al. [33] conducted a study with a cross-sectional design. A survey of 1015 RPNs in Manitoba was conducted to determine the predictors, prevalence, correlates, and distribution of vicarious trauma and burnout. The survey included the Maslach Burnout Inventory, the Traumatic Stress Institute Belief Scale, and a section on posttraumatic stress disorder (PTSD) symptoms. Seventy-nine percent of the respondents were female, and 20.2% were male. Emotional exhaustion was the highest in RPNs working in community services and acute care hospitals. Constant interruptions, burdensome responsibility, increased trauma work, depersonalization, and elevated vicarious trauma scores were linked with higher emotional exhaustion levels. With regards to vicarious trauma, 21% had persistent thoughts in relation to client trauma, and 30% experienced a heightened level of arousal. Client trauma is the exposure to a stressful experience that overwhelms a person's coping mechanism. Fifty-five percent of those involved in client trauma met one criteria of PTSD, and 48% responded that symptoms were troublesome to some degree. Lack of peer support and skills to deal with trauma could be rectified by increased education and team building. The RPNs in this study also reported a high level of personal accomplishment, which is associated with low burnout [33]. Personal accomplishment includes the perception that clients are improving and the RPNs have the skills necessary to help individuals with mental health disorders. The study is significant in that it mirrors the results of the studies in the preceding discussion concerning nursing quality of work life. Stress is evident in nurses working in mental health care and impacts the mental health of the caregivers. On the other hand, personal accomplishment was high amongst the RPNs, and this may decrease burnout. How this impacts client care requires further study.

Ryan-Nicholls [34] studied the repercussions of health reform in RPNs. Seven focus groups with RPNs from a diversity of health care settings took place in Manitoba over a nine-month period. The themes that emerged consisted of changes from institutional care to the community, variations in professional position, primary and secondary care and prevention, lack of provincial communication, and consistency amongst policies. Deinstitutionalization was considered to have largely impacted the practice setting of RPNs. This change led to an extension of their roles in many different settings including emergency departments, treatment centers, and acute psychiatry. RPN professional status has required a move to more autonomous roles within the primary care setting where professional competence is emphasized. Emphasis on health promotion and prevention is in contrast to the traditional approaches that focused on treating illnesses in institutionalized settings. Study participants described lack of consistency with mental health standards, protocols, and policies in the region. This was not conducive to the provision of mental health services [34].

In the same study, the RPNs became more familiar with the growing emphasis client-centered care and the need for mental health consumers to be involved in the decision making process. The transition from the traditional institutionalized care where decisions were made for the clients had left some RPNs feeling unprepared and concerned that the client may not choose what is best for them. The importance of engaging the family has become more apparent and this contrasts to the way care was provided in the past where family involvement was limited in the institutional setting [34]. The education of RPNs and RNs is essential to meet the changing approaches to caring for those with mental illness and the growing recognition of the impact of social determinants of health on the health and well-being of diverse populations. There is scant research that clearly indicates how the diverse educational preparation of nurses working in psychiatric care impacts the quality of care for persons with mental health disorders. Given the burden of mental illness in Canada there is need for more research that analyzes the psychiatric educational preparation of nurses in relation to accessibility to mental health care and therapeutic and treatment outcomes. Client-centered care requires advocacy and involvement with family and community beyond the confines of institutional care and this may require increased education specific to the changing dynamics of mental health care within the community setting.

According to Ryan-Nicholls [34], the shortage of RPNs is a concern of the existing RPNs. Previously, it was believed that deinstitutionalization would perhaps leave RPNs without jobs. Now, there are not enough RPNs to fill the vacant positions. In addition, participants in the focus groups discussed concern about the 2-year diploma program changing into the 4-year baccalaureate degree for RPNs, graduating only 15 students per year as compared to 60 students per year from the diploma program [34]. Therefore, the issues for RPNs parallel to the issues of the broader nursing workforce, where the shift to higher education has impacted the size of the nursing workforce, but at the same time the nursing graduates of today have increased knowledge to meet the current challenges of health care.

## 4. Discussion

The diversity between the organization of PMHN and education between the western provinces and the rest of Canada has connections with events of the past and intersects with gender, professionalization, and the predominating organizational culture. The historical analyses of nursing may foster additional inquiries that may benefit nursing knowledge through learning from past approaches and gaining new perspectives.

Overall PMHN in Canada is challenged to further itself to meet the lack of accessible mental health services. There is evidence that nurses are stressed, and there is a need to enhance a coordinated national approach for advanced degrees in PMHN. The lack of a uniform approach to PMHN education in Canada has consequences for the further development of PMHN and may generate barriers to further PMHN to meet the growing mental health care challenges. The implementation of standards by the Canadian Federation of Mental Health Nursing is brought to the discussion here in order to explain how standards are an important way to address the diverse forms of PMHN education. The standards foster an overarching educational approach for PMHN that enables quality PMHN practice. In 2006, the third edition of the Canadian* standards for psychiatric-mental health nursing* was released in an effort to prompt nurses to adopt the standards into daily practice and further nursing reflection on their work [35]. The latest standards were developed after consultation with Canadian consumers of mental health across Canada. Beal et al. [36] acknowledge that systemic issues, that is, labeling, stigma, caregiver, and treatment role, affect PMHN but emphasize the need for nurses to know their clientele to foster therapeutic relationships. Systemic factors including workforce size, workload, violence in the workplace, nursing scope of practice, and accessibility to PMHN may have important implications for PMHN and their daily practice. PMHN education strives to produce nurses whose practice meets or exceedes the standards [36]. However, systemic factors must be addressed to foster PMHN's delivery of high quality care that are consistent with the* standards for psychiatric-mental health nursing.* Furthermore, as an influential and strong stakeholder that can affect change in mental health care, PMHN may benefit from a uniform educational process throughout Canada.

Also of concern is the mental health of nurses who experience high stress. As nurses are mostly women and nurses form the largest group of health care providers, the ramifications for the health and productivity of the Canadian society are especially disconcerting if the majority of nurses are experiencing reduced quality of work life. As Wall [20] and McGibbon et al. [21] point out, a sociological paradigm enables the discussion of sensitive issues including the gendered impact over nursing and the need for research from a perspective that views critically the influence of gender on quality of nursing work life. The labour divisions in organizations where nurses practice are entrenched in hierarchal power struggles that undermine nursing knowledge and autonomy and contribute to poor quality of work life and stress.

### 4.1. Limitations of Review

There were limitations encountered in this review. Although several studies were found in relation to the history of PMHN for the western provinces and Ontario, no studies were located that convey the full PMHN history of eastern Canada. While there were government documents concerning the quality of work life for all nurses, it was difficult to abstract information specific to PMHN from these documents. For instance, although the NSWHN found a high incidence of depression amongst nurses, the percentage of nurses in PMHN experiencing depression was not given. It is acknowledged that the whole puzzle of what PMHN is like in Canada is not complete. Furthermore, as NPs are becoming more established within the Canadian health care system there is a need for increased research that reveals NP mental health care initiatives and activities. At present, there is a lack of research pertaining to Canadian NPs employed in mental health and their quality of work life. Despite this, the findings reported here depict important points regarding the issues that concern all of PMHN and how they impact the provision of mental health care for Canadians.

### 4.2. Implications for Nursing Practice

While NPs have made significant progress in achieving prescriptive authority in the area of primary care, minimal movement has occurred regarding the feasibility of NPs specializing in mental health. In Canada, there are no educational programs or legislated provisions for NPs who wish to specialize as a psychiatric NP or who already have extensive experience providing mental health care [37]. The existing situation for NPs practicing within mental health care settings is hampered by the absence of recognition for the psychiatric mental health nurse practitioner (PMHNP) in Canada. Prescriptive authority can only be obtained in Ontario through registration in the extended class in the designated specialties, NP paediatrics, NP-Primary Health Care and NP-Adult. The educational programs that prepare NPs for these specialties focus on the medical and physiological aspects of the specialties with limited content on mental health [37]. In 2011, open prescribing became possible for Ontario NPs. The Nursing Act, 1991, no longer stipulates the prescribing of only listed medications by NPs [38]. With the exception of medications under the Controlled Drugs and Substances Act, NPs are now able to prescribe medications commonly used in mental health care. This allows NPs to further their care for clients needing mental health care; however, it may also point to the need for comprehensive mental health training for NPs. It beckons further exploration if the existing mental health nursing programs offered throughout Canada could enable the development of PMHNP programs that would enable RNs or RPNs and NPs to obtain the competencies of the PMHNP. Communities with insufficient mental health care resources may be better served by nurses with an expanded scope of practice with specialized mental health education. In addition, although there have been changes to legislation, specifically regulation 965 of the Public Hospitals Act, that gives admitting and discharging privileges to NPs; changes to the mental health act have not been made and regulated forms enabling admission to a hospital for psychiatric assessment cannot be completed by Canadian NPs [37]. When the NP is the person's main care provider, it would seem prudent that the NP should be involved in decisions concerning psychiatric admission and discharge.

The variability of PMHN in Canada presents both challenges and opportunities to the advancement of PMHN education. Given the challenge for increased accessibility to mental health care services and treatments, the development of already existing nursing programs in Canada could bond by striving to achieve the same psychiatric educational nursing standards. To further mental health care it may be possible for all nursing programs to uphold the same standards for psychiatric nursing education. The CNA motioned a resolution in 2005 to include RPNs as it was recognized that a separate national level for RPNs would hinder professional nursing practice and the power for Canadian nurses to advocate for change [39]. Amending the division between RPN and RNs and to enable certification and eligibility of CNA membership for both groups may strengthen the Canadian PMHN workforce. Gallop [40] considers how the education system in Canada lacks prospects for nurses working in mental health to obtain advanced degrees in PMHN, although, as previously mentioned, there is new potential for RPNs and nurses to obtain master degrees specializing in mental health nursing west of Ontario. As mental health care is no longer confined to institutional settings and mental health training is pertinent to all areas of health care, the necessity for advanced mental health education is relevant to all health care settings where nurses practice. Nursing needs to facilitate advanced education in mental health so that people within the primary care setting also benefit from the knowledge and expertise of nurses who have additional mental health education. Bridging programs or additional educational opportunities for psychiatric nurses wishing to broaden their knowledge specifically to prescribe psychiatric treatments or medications may be helpful. Partnerships between provincial nursing bodies to foster national standards for PMHN education and bridging programs to a PMHNP program if developed may represent new opportunities for all Canadian nurses. The report entitled “*Canadian nursing education in Canada statistics,” 2009-2010 *[41], conducted by the Canadian Nurses Association (CNA) and the Canadian Association of Schools of Nursing (CASN), reveals an increase of 64.3% from 2009 to 2010 for graduates for doctoral programs [41]. In 2010, 47.6% of NPs had a master's, and 0.8% of NP had a doctorate degree [4]. More nurses and NPs are acquiring advanced degrees. With this trend for advanced education, it is likely that mental health education for all nurses and nursing research pertaining to mental health and PMHN will flourish. Advanced PMHN education offers possibilities to further mental health care for Canadians.

## 5. Conclusion

PMHN in Canada must take action to meet the goals as set out by the Mental Health Commission of Canada. National standards for psychiatric nursing education for all Canadian nursing education programs may positively impact mental health care. Advanced education for PMHN including the development of a Canadian PMHNP program may further the accessibility of psychiatric treatments. Psychiatric mental health education for all nurses will complement primary health care and the provision of mental health care in general hospitals and community settings. Efforts to unite and form partnerships with the varying groups providing PMHN at a national level may empower PMHN as a powerful stakeholder in mental health care reform. Also, the mental health of nurses in light of their quality of work life is a red flag for all leaders in the Canadian health care system. There is a need to understand the circumstances of nurses with regards to occupational stress and barriers to advanced education in mental health nursing. Although the full picture of what Canadian mental health nursing looks like today cannot be fully realized by this synthesis, important issues facing PMHN have been identified through the analysis of history and application of a critical and gendered sociological lens. Another chapter on the evolution of Canadian PMHN has yet to be written; hopefully, it will entail how PMHN works together with all stakeholders equally to provide the best care possible.

## Figures and Tables

**Table 1:** Studies using a gendered, critical and sociological perspective relevant to PMHN.

Author and date	Purpose	Design/methodology/approach	Province	Sample size	Findings
Wall (2010) [20]	To critique nursing research on nursing practice environments using a critical sociological perspective To propose a paradigm for future research	Review of research on nursing practice environments Sociological concepts are linked to variables in the nursing literature	Refers to nursing in general within the Canadian context	NA	Nurses' job satisfaction can be linked to gender knowledge, professionalization, and organization not just management concerns

McGibbon et al. (2010) [21]	To reformulate the nature of nursing stress with regards to context	Interviews, participant observation, and focus groups with pediatric ICU nurses Theoretical perspective uses Smith's critical sociological frame of institutional ethnography	Study occurs within the province of Ontario	23 nurses	Six main forms of stress—emotional distress, constancy of presence, burden of responsibility, negotiating hierarchical power, engaging in bodily caring, and being mothers, daughters, aunts, and sisters

**Table 2:** History, gender, and educational implications for Canadian psychiatric mental health nursing

Author and date	Purpose	Design/methodology/approach	Province	Sample size	Findings
Tipliski (2004) [23]	How Canadian psychiatric nursing developed into two entirely different models?	Historical analysis—case studies in the provinces of Ontario, Manitoba, and Saskatchewan	Ontario, Manitoba, and Saskatchewan	Not applicable (NA)	PMHN development can be understood through psychiatry's authority in the context of the gender limitations traditionally imposed upon women and nurse leaders Ontario nurse leaders took control of psychiatric nursing, whereas in Manitoba and Saskatchewan they did not

Dooley (2004) [24]	Historical account of Manitoba's distinct mental health nursing	Labour history—historical analysis—oral testimony of Manitoba's nursing graduates from the 1930s	Manitoba	NA	Manitoba—distinct class of PMHN giving rise to the regional differences where psychiatric nursing education is not integrated into general nursing education

Hicks (2008) [25]	Examination of factors that lead Manitoba to adopt the western style of PMHN and RPN class	Genealogical analysis from archives, interviews, and secondary sources	Manitoba	NA	RPN in Manitoba is a political, contingent development that will evolve

Hicks (2011) [27]	Historical account of professionalization of Manitoba's RPNs	Historical analysis from archives and secondary sources	Manitoba	NA	RPN profession in Manitoba arose to fill inadequately staffed mental hospitals then developed into a specialized workforce

Boschma et al. (2005) [9]	How gender shaped training, work opportunities, and professional identity of PMHN?	Social history method of analysis—interviews with questionnaires of PMH nurses who practiced between 1939–1990	Alberta	34 women, 9 men	Alberta's PMHN professional identity shaped by gendered care ethic

Boschma (2012) [26]	Exploration of how psychiatric nurses understand and create their role in Alberta	Historical analysis, case study, and oral history interviews	Alberta	NA	Alberta's present community mental health service is evolved and transformed from the prior institutional practice

**Table 3:** Canadian mental health psychiatric quality of nurses work life.

Author and date	Purpose	Design/methodology/approach	Province	Sample size	Findings
Robinson et al. (2003) [33]	To examine prevalence, distribution, correlates, and predicators of vicarious trauma and burnout among registered psychiatric nurses (RPNs) in Manitoba	Survey contained the Maslach Burnout inventory, the Traumatic Stress Institute Belief Scale (TSIBS), and a section on PTSD	Manitoba	295 surveys returned, response rate of 29%	RPNs experiencing high levels of emotional exhaustion and higher levels of personal accomplishment. No significant difference on the TSIBS

Ryan-Nicholls (2004) [34]	To address impact of health reform on RPN practice	Focus groups	Manitoba	33	Increase in independence. Insufficient preparation for new roles, current, and future shortage of RPNs

Statistics Canada (2006) Findings from the 2005 National Survey of the Work and Health of Nurses [31]	To assess work and health of Canadian nurses	Survey	All provinces	19000 nurses including RNs, licensed practical nurses (LPNs), and RPNs	Mental and physical health problems of nurses linked to work stress, low autonomy, shift work, poor physician-nurse relationships, low support, and lack of respect

Health Canada (2002): *“Our health, our future: creating quality workplaces for Canadian nurses, final report of the Canadian Nursing Advisory Committee"* [29]	To improve quality of nursing work life	6 researches & information projects	All provinces		(i) Paucity of data on licensed practical nurses and RPNs (ii) 51 recommendations (3 categories): (1) implement conditions to resolve work load, overtime, and absenteeism and decrease nonnursing tasks; (2) create professional practice environment in leadership, education, and prevention of violence and abuse; (3) monitor workplaces and health of nurses, accreditation, and research and disseminate information to keep nurses informed

Maslove and Fooks (2004) [30]	To learn what actions to implement recommendation by the Canadian Nursing Advisory Committee (CNAC) made in the 2002 *“Our health, our future: creating quality workplaces for Canadian nurses"* report	Scan of web sites, letter to stakeholders to learn what had been done, interviews with key informants, and presentation with 14 representatives from nursing stakeholders in Ottawa	All provinces	94 stakeholders, 14 key informants	Increased number of education seats for RNs, LPNs, and RPNs, workload measurement systems, increased number of full time positions, nurse mentors, and flexible scheduling—progress not uniform but concentrate in acute care rather than community; responsibility to carry out recommendations is unclear, need for CNACs, leadership position, funding, survey, accreditation, research to identify problems

Health Canada (2007): *“The working conditions of nurses: confronting the challenges"* [32]	To examine research on the state of Canada's nurses and implication for a larger health care system	Bulletin	All provinces	NA	Workplace and workforce issues require collaboration and government involvement
